# Impact of an age, kidney and liver function adjusted sedation protocol in critically ill patients

**DOI:** 10.1186/cc12319

**Published:** 2013-03-19

**Authors:** S Haddad, H Tamim, C Gonzales, A Rishu, A Deeb, Y Arabi

**Affiliations:** 1 King Abdulaziz Medical City, Riyadh, Saudi Arabia

## Introduction

Daily sedation interruption and protocol implementation have been recommended to reduce excessive sedation; however, their use has been inconsistent. We hypothesized that the use of an age, kidney and liver function adjusted sedation protocol would be associated with reduced doses and improved outcomes compared with a standard protocol.

## Methods

This was a prospective cohort study comparing 3 months of a standard protocol (control group) with 3 months of an adjusted protocol (intervention group). In the adjusted protocol, patients were divided into three categories: category 1 (age <60 years, and normal kidney and liver function), category 2 (age = 60 to 70 years, or moderate kidney or liver function impairment), and category 3 (age >70 years, or severe kidney or liver function impairment). The upper limits of analgesics and sedatives doses were determined by age, and kidney and liver function, being lowest in category 3, and lower in category 2 than category 1. All consecutive adults mechanically ventilated patients who required infusion of analgesics and/or sedatives for >24 hours were included in the study. We compared the main outcomes of both groups including average daily doses of analgesics and sedatives; average Sedation-Agitation Scale (SAS), pain and GCS scores; mechanical ventilation duration (MVD); sedation-related complications during ICU stay; ICU and hospital length of stay (LOS), and ICU and hospital mortality.

## Results

Two hundred and four patients were included in the study (control group = 105; adjusted protocol group = 99). There was no difference in baseline characteristics between the two groups. The adjusted protocol group, compared with the control group, received significantly lower average daily doses of fentanyl (2,162 ± 2,110 μg vs. 3,650 ± 3,253 μg, *P *= 0.0001), nonsignificant lower average daily doses of midazolam and dexmedetomidine, and a trend toward higher average daily doses of propofol. Pain score was higher in the adjusted protocol group (0.98 ± 0.72 vs. 0.16 ± 0.35, *P <*0.0001) with no difference in SAS or GCS scores. Sedation-related complications during ICU stay were not different between the two groups; however, agitation (SAS = 5) was less frequent in the adjusted protocol group (3% vs. 30%, *P <*0.0001). ICU mortality was significantly lower in the adjusted protocol group (18% vs. 36%, *P *= 0.004) with no significant differences in MVD, ICU and hospital LOS, and hospital mortality.

## Conclusion

The use of an age, kidney and liver function adjusted sedation protocol is associated with lower doses of analgesics and sedatives, lower risk of agitation and lower ICU mortality.

